# Family ownership and control as drivers for environmental, social, and governance in family firms

**DOI:** 10.1007/s11846-023-00631-2

**Published:** 2023-03-07

**Authors:** Jiamu Sun, Massimiliano Matteo Pellegrini, Marina Dabić, Kai Wang, Cizhi Wang

**Affiliations:** 1 School of Economics and Management, Beijing Jiatong University, Beijing, China; 2 Department of Management and Law, Tor Vergata University, Rome, Italy; 3 Faculty of Economics and Business, University of Zagreb, Zagreb, Croatia; 4 University of Dubrovnik, Dubrovnik, Croatia; 5 School of Economics and Business, University of Ljubljana, Ljubljana, Slovenia; 6 College of Business Administration, Capital University of Economics and Business, Beijing, China

**Keywords:** ESG criteria, Family involvement, Family ownership, Family control, Socioemotional wealth theory

## Abstract

Sluggish market demand can deteriorate the financial situation of a company and affect a shareholder’s decision to adopt environmental, social, and governance criteria (ESG). According to the socioemotional wealth theory, family firms place significant emphasis on sustainable development and long-term orientation, but this emphasis can be either internally or externally driven according to the type of involvement chosen by the owning family. Therefore, this study uses listed family firms to explore the relationship between different types of family involvement (i.e., family ownership and control, the influence of market competition, and the institutionalisation level of the environment in which a firm decides to pursue ESG criteria). We performed a multivariate regression analysis on a sample of 1,151 Chinese companies to test these relationships and found that both family ownership and control are positively related to ESG scores. Market competition negatively moderates the influence of both family ownership and control on the adoption of ESG criteria. Moreover, the influence of family control is negatively moderated by the institutional environment. Thus, types of family involvement seem to be relevant for the firm’s engagement with ESG criteria.

## Introduction

Since the addition of environmental, social, and governance (ESG) criteria to the Principles of Responsible Investment (PRI) of the United Nations, ESG disclosure has become mandatory in many countries, particularly in Europe and Asia (Friede 2019; Daugaard 2020; Gillan et al. 2021). ESG criteria are composed of a system of comprehensive indicators divided into three categories: environmental management, social responsibility, and corporate governance (Eccles and Viviers 2011; Nirino et al. 2021; Xiang et al. 2021). These criteria are adopted primarily by financial institutions when making investment decisions and setting out principles, such as removing or adding financial products to a blacklist (i.e. businesses working with weapons or cigarettes) or when investing money in financial products that meet certain standards (Friede 2019; Daugaard 2020; Widyawati 2020). For these reasons, nowadays these criteria are widely used and recognised not only when disclosing and reporting but also when making decisions regarding firm strategies and operations (Clementino and Perkins 2021; Gillan et al. 2021; Nirino et al. 2021). Yet, commitment to ESG criteria can differ in terms of intensity: One approach may be more pervasive, allowing ESG criteria to truly guide a company’s strategies and operations, while another may be more limited, only passively disclosing ESG information (García-Sánchez et al. 2020; Clementino and Perkins 2021). This higher or lower level of engagement with ESG criteria usually results in better (lower) ESG scores that several independent agencies (e.g. Thomson Reuters) assign to listed companies to clearly display engagement (Daugaard 2020). The academic literature has also indicated that decisions pertaining to levels of engagement with ESG criteria are mainly influenced by deliberations regarding shareholder assembly (Diebecker and Sommer 2017; Rossi and Harjoto 2020).

Considering the specific and relevant topics that influence shareholders’ assembly, families that own a business should be examined even more, especially considering that this coalition has been shown to emphasise sustainable development and long-term orientations (Chrisman et al. 2012; Zellweger et al. 2012b; Kraus et al. 2018, 2020a; Rovelli et al. 2022). The willingness to maintain ownership over generations is one of the main characteristics used to identify family firms. As such, the goal-setting process of a family business should naturally align with ESG criteria. This can facilitate sustainable development and, in turn, favour long-term orientations and prosperity for the business over time (Villalonga 2018; Casado-Belmonte et al. 2021; Cordeiro et al. 2021; Fritz et al. 2021). For example, the reputation, identity, and legacy of a family firm and the perception of the local community are all paramount considerations for a family firm, and they all exist within a non-economic sphere of goals (Chrisman et al. 2012; Lazzarotti et al. 2020; García-Sánchez et al. 2021). In this sense, attention should result in an improvement in sustainable practises and a greater use of ESG criteria. This relationship is even more important for large family firms (Peng et al. 2018; Williams et al. 2018). Survival and renovation/defence of the competitive advantage of large companies are tied to the consistent support and participation of all stakeholders (Gomez-Mejia et al. 2011; Hafner 2021). In turn, the possibility of passing ownership to future generations of family members can occur only if the company is committed to implementing strategies and practices to ensure balanced growth, where economic and non-economic considerations are simultaneously met (Gomez-Mejia et al. 2011; Chua et al. 2015; Tiberius et al. 2021). Through transactions, investments, and collaborations, large companies interact with and impact several external stakeholders involved with a family firm. Involvement with a larger audience of external stakeholders requires an intensive commitment of resources to be satisfied and thus demands a more discerned allocation and balance between family and non-family goals (Chrisman et al. 2012; Mariani et al. 2021; Garcés-Ayerbe et al. 2022). For example, a larger number of employees could imply stronger union power, and thus more efforts could be made to improve social welfare. A large amount of business in a certain area leads to a higher impact on local communities through, for example, local tax contributions and workforce absorption. This leads to further collaboration with local communities (Sacristán-Navarro et al. 2011; Ortas et al. 2019). However, recent studies have explored how family involvement can be detrimental to non-economic goals, and thus sustainable development, for the sake of firm performance. That is, when firm performance is lower than expected, the owning family may make more market-orientated decisions, unbalancing the satisfaction of non-economic goals. In this way, the company tends to behave not as a family-owned company but as one with more market-driven logic (Marques et al. 2014; Tiberius et al. 2021). Therefore, we can find positive and negative influences of family involvement in a business in terms of social responsibility (Mariani et al. 2021) and environmentally friendly practices (García-Sánchez et al. 2021). In summary, family firms can be found to focus on sustainable practices (e.g. Agostino and Ruberto, 2021; Clauß et al. 2022). These inconsistent findings on family involvement and its impact on ESG and sustainable practises suggest the need for further research. Therefore, we raise the following research question: How does family involvement affect the adoption of ESG criteria and ESG scores?

To answer this question, the present study adopts the socioemotional wealth theory (SEW) thanks to the attention paid to non-economic goals and their pursuit (Gómez-Mejía et al. 2007, 2011). We propose that, in large family firms, the engagement and satisfaction of stakeholders through the adoption of ESG criteria is paramount to preserving SEW capital endowments (Cordeiro et al. 2021; Fritz et al. 2021; Clauß et al. 2022). However, the inconsistent findings so far presented in the academic literature may be related to an unclear awareness of the type of influence that a family can exert over a shareholder assembly. The first type of involvement, termed ‘family ownership’, refers to the overall level of cash flow rights throughout the chain(s) of ownership compressively attributed to a single family (Villalonga and Amit 2006). The second type is the overall power that a family can exert over the assembly. We term this ‘family control’, because a family can make decisions directly or indirectly through chain(s) of control. As these involvements are different in nature (Anderson and Reeb 2003; Chrisman et al. 2012), the attention paid to a family’s intention to maintain cross-generational control, manage stakeholders’ satisfaction, and, in turn, maintain the engagement with ESG could also change (Chrisman et al. 2012; Kraus et al. 2016; Wang et al. 2020; García-Sánchez et al. 2021). More recent SEW studies have begun to reveal that, although non-economic goals are central to family firms, especially in times of economic constraint, the trade-off between non-economic and economic goals becomes more difficult to find, and the balance can be broken (Kalm and Gomez-Mejia 2016; Swab et al. 2020; Garcés-Ayerbe et al. 2022). For example, when market competition increases, this can lead to companies prioritising economic goals over pursuing non-economic goals, and in turn, the family coalition may sacrifice ESG practices to improve competitiveness (Berrone et al. 2012; Ratten et al. 2021). However, the institutional environment can also be a contextual factor that constrains family decisions, as the bigger the power exerted over a firm, the more regulations are enforced against the dominant coalition (Kariv and Coleman 2015; Garcia-Sánchez et al., 2021). For example, family-controlled firms may have a more rigid level of corporate governance compliance by, for example, increasing information asymmetries and imposing stricter rules and regulations when taking strategic actions. These can also influence the adoption of ESG criteria (Lien et al. 2016; García-Sánchez et al. 2021).

Consequentially, we conducted a multivariate regression analysis using panel data (2015–2019) from listed firms in China to test our hypotheses, leading to several inspiring findings. First, we found a positive effect of both family ownership and control on the adoption of ESG criteria. This is consistent with the pursuit of non-economic goals postulated by the SEW theory. However, when competitiveness needs to be improved, a family may sacrifice ESG engagement. Moreover, institutionalised environments can lead to more limited strategic actions towards ESG practices.

The structure of the paper is organised into five sections, including this introduction. Section 2, using SEW theory, describes how a family influences engagement with ESG criteria and scores through moderation mechanisms. Section 3 describes the research methods, describing the data collection procedure, the variable measurements, and the models used. Section 4 is dedicated to the empirical results that confirm our hypotheses. Section 5 provides a discussion of these results and conclusions, including contributions, limitations, and possible future research directions.

## Theory and hypotheses

### ESG criteria

Since their introduction into the PRI of the United Nations, ESG criteria have been largely recognised by listed and large firms as a major ‘tool’ to disclose environmental management, social responsibility, and other non-financial information (Gillan et al. 2021). Traditionally, studies focused on ESG have mainly adopted a financial investment perspective. ESG criteria are made up of indicators belonging to three spheres: environmental management (E) (e.g. disclosure about carbon emissions and pollution control strategies), social responsibility (S) (e.g. strategies to improve community welfare and promote stakeholders’ health and safety), and corporate governance (G) (e.g. independence and diversity of the board of directors) (Friede 2019; Daugaard 2020). Financial institutions use these indicators to better interpret annual reports and other information disclosed by listed firms and to rate firm performance according to each category (E-, S-, and G- sections, respectively). Several agencies have issued ESG scores to quantify the ESG engagement of listed companies. For example, Thomson Reuters created the *ASSET4* index, while MSCI issued the *MSCI KLD 400 Social Index* to rate firms with ESG scores, helping investors get used to referring to these scores when making their investment decisions (Daugaard 2020). Meanwhile, these financial institutions have introduced financial products, such as funds, that are based on ESG criteria. When raising funds that meet the ESG criteria, there are two principles: positive and negative screening (Widyawati 2020). Positive screening occurs when investors use ESG scores to select the best options and thus invest only in financial products that meet certain standards. For example, ESG climate change is one of the *MSCI* funds that invests only in businesses with low carbon emissions. Negative screening instead refers to the creation of ‘blacklists’ of businesses, certain businesses or industries (such as those involving drugs, weapons, or cigarettes) that will not be considered for investment. For example, *EURO STOXX 50* is a financial index based on market capitalisation which groups the top 50 liquid stocks in the Eurozone. When choosing these 50 firms, the adopted investment principle involved negative screening, which excluded not only firms of a certain industry (e.g. weapons, thermal coal, military contracting, and tobacco), but also those with low ESG scores.

An increasing number of institutional investors have committed to the PRI and follow the ESG criteria. Because of this, ESG scores are becoming an institutional signal to increase financial ability to raise capital on regulated capital markets (Ortas et al. 2019; Widyawati 2020). Thus, indirectly, firm behaviours are influenced and forced to conform through the institutional environment and its pressure (Kordsachia et al. 2022). Therefore, the adoption of ESG criteria can result in high pressure for institutional isomorphism and thus serve as an externally driven motivator for adoption (Diebecker and Sommer 2017). However, many organisations have found it advantageous for them to adopt ESG criteria for internal reasons (e.g. if the personal or organisational values of a shareholder or a shareholder coalition are aligned with the fundamental purposes of ESG criteria). This may be the case when shareholders place emphasis on long-term orientation interests such as the appreciation of the stock value of shares (García-Sánchez et al. 2020). Furthermore, shareholder judgment can also positively influence ESG criteria if these become fairly common industrial production standards and are proactively adopted in firm operations and strategies (Ortas et al. 2019).

Whatever the motivation to adopt ESG criteria, many empirical studies have supported the idea that ESG scores are beneficial to firm performance, especially in terms of stakeholder engagement (Fritz et al. 2021). In line with the development of an institutional market, many external stakeholders have started adding ESG criteria to their contract terms. A typical context in which to study such an influence is supply chain management. Here, ESG criteria favour focal firms in maintaining relationships with upstream and downstream firms along the distribution or production channel (Mukandwal et al. 2020). However, ESG criteria signify a balanced governance model and are thus used to set pricing strategies. For example, in the field of commercial mortgages and real estate investments, the cost of debt for secondary market transactions seems lower for environmentally certified buildings (Eichholtz et al. 2019). However, these criteria can also directly improve firm performance (Widyawati 2020). ESG criteria balance different interests for internal or external groups and are a good standard for improving corporate governance models, yielding improved performance (Gillan et al. 2021). Irrespective of whether ESG criteria facilitate these results directly or indirectly, they are not only concerned with economic and business goals but also the means through which these results are obtained, increasing attention to non-economic considerations and more sustainable practices (Ortas et al. 2019; Widyawati 2020). However, the pursuit of ESG criteria may be challenged by turbulent macroeconomic contexts and poor firm performance (Lee et al. 2016; García-Sánchez et al. 2021). The rising prices of energy and raw materials can alter the shareholders’ resolution to pursue non-financial and sustainable goals. Therefore, this study also draws attention to the factors that stimulate shareholders’ decisions to pursue non-economic goals and ESG scores.

### Socioemotional wealth theory, family involvement and ESG

The SEW theory stems from the behavioural aspects of the traditional agency theory, emphasising relational criteria in governing a firm (Gómez-Mejía et al. 2007). Later studies (e.g. Berrone et al. 2012) summarised the features of the SEW theory and organised them into five dimensions: (i) Family influence concerns the tendency of a family to foster consistent family-like values into the business during a family-to-firm transfer; (ii) the identification mainly refers to the conviction that the family reputation is intimately and indissolubly bound to business success and reputation (Chrisman et al. 2012; Naldi et al. 2013); (iii) binding social ties concerns the tendency of family firms to have strong relationships, both internally, with non-family employees, and externally, with suppliers and business partners. More generally, however, these binding ties are also created with society (e.g., through community development initiatives and with other institutional entities, such as commercial associations, research centres, and financial institutions) (Wang et al. 2021; Bichler et al. 2022); (iv) emotional attachment can be used to explain the commitment that a family has to the business, investing resources and time in the company and its development, as this forms part of their family history (Kraus et al. 2018; Fritz et al. 2021), and (v) the renewal of family bonds refers to long-term orientation and the intention of succession, implying that a family wants to maintain control over the business for the next generation of family members (Lazzarotti et al. 2020).

According to the SEW theory and its dimension, family firms tend to preserve non-economic goals, which are naturally related to ESG criteria and sustainable development. This natural correlation can be explained through two aspects: inward- and outward-orientated. Within an organisation, family identification and emotional attachment may drive family firms to support the adoption of ESG criteria. In particular, family firms tend to shape an organisational environment to foster reciprocal altruism, a condition that stresses the need for trust between employees, bridging social ties among employees, and fostering a collective culture (Kariv et al. 2009, 2010; McGrath and O’Toole 2018). A collective and mutual-trust culture helps mitigate any conflicts of interest within firms (Schulze et al. 2003). This is an important indicator of the ESG corporate governance sphere. This also improves employee satisfaction (Zahra 2012), and employee feedback is one of the main aspects pertaining to human rights, which belongs to the ESG social responsibility sphere.

Outside of the organisation, the willingness to maintain the binding of social ties and renewing family bonds drives business-owning families to commit resources to social ties with key stakeholders and customer relationships (García-Sánchez et al. 2021; Rovelli et al. 2022). The intention to preserve SEW drives family firms to simultaneously meet the interests of different stakeholders and support the development of the community (Cordeiro et al. 2021). On the one hand, protecting stakeholder interests and community welfare can directly increase scores in the ESG social responsibility sphere. On the other hand, carrying out corporate social responsibility initiatives can further promote the willingness of a firm to actively invest in environmental management, or at least prevent environmental scandals (Kraus et al. 2020b). For example, when industrial associations, suppliers, communities, or those on social media start to appeal to the adoption of ESG criteria, the intention to preserve family reputations and binding social ties can drive family firms to increase ESG scores, such as pollution control and waste management.

If SEW preservation naturally aligns family decisions with ESG criteria, the different extents to which a family can influence the shareholders’ assembly can interfere with this trend (Casado-Belmonte et al. 2021). The first type of involvement refers to family ownership (i.e. the overall quota of cash-flow rights possessed by a family). From a governance and shareholders perspective, this influence may be closer to a non-family or large coalition ownership perspective in managing the firm, and it usually occurs in long-lived and established family firms that have already reached the third or more generation of ownership (Anderson and Reeb 2003; Villalonga and Amit 2006). This is because the family in this case is interested in preserving their financial investment and cross-generational ownership, rather than directly controlling the firm’s operation and strategies. Owning without directly managing the actual operations of a company implies a decrease in emotional attachment, but this is a ‘genetic’ concern for the preservation of the SEW endowment and is mainly guided by the outward motivations previously mentioned. It is less concerned with inwardly orientated SEW preservation. Family ownership can lead to a more rational approach to management, such as the hiring of independent directors, the adoption of a professional top management team (Sacristán-Navarro et al. 2011), and the willingness to share and disclose more information to stakeholders (Cordeiro et al. 2021), in awareness of the fact that reputational issues and proper governance models are vital in ensuring the company’s survival. In summary, the engagement with the ESG criteria of a family-owned firm is mainly externally orientated, and a large number of stakeholders and shareholders need to find satisfaction along the chain of ownership. More professionals are hired in top management teams to complement the skillsets of family members (Kalm and Gomez-Mejia 2016). For this reason, alignment with the ESG criteria seems to be more natural. Therefore, we can postulate the following:

#### *Hypothesis 1*

Family ownership is positively related to the ESG score of a firm.

Other considerations arise with regard to ‘family control’. This element is the total amount of power that a family can exert over a shareholder assembly. In particular, this control relates to the ‘formal/direct ownership’ and to the whole chain(s) of ownership and the voting rights ascribable and indirectly to the family. This control influences the decisions of the assembly, since the family can also reinforce its strategic orientation through delegates and cumulative participation in other companies. The influence of family control differs from that of the family ownership, as a deeper involvement in firm operations is involved. This also implies a stronger emotional attachment to the company, a situation that is more common among first- or second-generation family members (Anderson and Reeb 2003). Family control suggests a stronger family influence in fostering consistent family-like values and reciprocal trust in the organisational culture. This may also arise when making decisions about the adoption of ESG criteria (Lien et al. 2016). Therefore, family control seems much more internally motivated when pursuing SEW preservation. Therefore, this internally driven motivation can raise some questions. On the one hand, especially when the business is so large that it is listed on a stock exchange market, forward-thinking families may recognise that cross-generational succession and growth can only be ensured through balanced and sustainable development and balanced governance models. As discussed, not complying with ESG criteria can be extremely harmful for a company and undermine its future survival (Clauß et al. 2022). In several of its dimensions, SEW theory implies that family firms should adopt naturally sustainable practices and comply with ESG criteria (Garcés-Ayerbe et al. 2022). For example, after examining emotional attachment and family-like firm, researchers have found that the employees of family-controlled firms have better social welfare and working environments (Villalonga 2018). Thus, it can be hypothesised that.

#### *Hypothesis 2a*

Family control is positively related to the ESG score of a firm.

In contrast, internally driven SEW preservation can also have negative consequences in relation to ESG engagement brought about by family control. From this internal perspective, the engagement with ESG is higher when the personal and organisational values pursued by shareholders align with the fundamental purpose of ESG. For this reason, some families may underestimate the importance of ESG criteria, especially in terms of promoting the survival of the business and thus reducing the actual possibilities to transfer the ownership rights to the new generations. This would hamper the engagement with ESG criteria (Gómez-Mejía et al. 2011). For example, from a negative perspective, SEW preservation may also lead to strategic inertia by extensively relying on strong ties that can limit information sources and creativity (Berrone et al. 2012), over-emphasising private relationships with stakeholders without considering costs (Swab et al. 2020) and excessively considering family relationships rather than objectively evaluating family members’ abilities (Kalm and Gomez-Mejia 2016). Not least, even if often considered a unique entity in the decision-making process, a family is formed by several family members with different powers, influences, and goals (Wang et al. 2021). This may result in a different perception of SEW motivations, thus negatively impacting ESG engagement. For all these reasons, a negative relationship should not be ruled out. Thus,

#### *Hypothesis 2b*

Family control is negatively related to the ESG score of a firm.

### Moderating effect of market competition

Both the preservation and firm competitiveness need to be of interest to the family through both types of involvement, be it be ownership or control (Kalm and Gomez-Mejia 2016). Current SEW studies have found that the preservation of SEW endowments should be balanced to consider economic and performance goals (Swab et al. 2020). Preserving SEW endowments is beneficial when it comes to transgenerational sustainability (Zellweger et al. 2012a), fostering goal congruency amongst the top management team (Campopiano and Rondi 2019) and maintaining strong ties with external stakeholders (Nason et al. 2019).

However, business-owning families cannot forget about the competitiveness of a firm (Chua et al. 2015) and its intensity can alter the family decisions. There is tension and a trade-off between non-economic goals (SEW endowments) and economic goals (firm competitiveness) in committing resources (Chua et al. 2015). Preserving SEW endowments may come at the cost of competitiveness, or, vice versa, competitiveness may require sacrificing resources from SEW capital. Although family firms tend to balance this trade-off, an increase in competitive pressure or a worsening of economic conditions may draw more attention and require more resources to implement a market response and restore competitiveness (Kalm and Gomez-Mejia 2016). In wake of the COVID-19 outbreak, geopolitical tensions and the related increase in material costs and financial burdens, firms tend to allocate more resources to firm competitiveness, ‘relaxing’ their commitment to their environment (Ratten et al. 2021). Therefore, if the SEW endowment is depleted, so is the commitment of family firms to ESG criteria, worsening a previous commitment (H1 and H2a) or exacerbating an already disputable situation (Hp2b). Hence, we propose the following hypothesis:

#### *Hypothesis 3a*

Market competition negatively moderates the relationship between family ownership and the firm’s ESG score.

#### *Hypothesis 3b*

Market competition negatively moderates the relationship between family control and the firm’s ESG score.

### Moderating effect of institutionalisation

ESG scores have become an institutional signal that is highly by both regulatory and financial institutions, which encourages companies to adopt ESG criteria. The institutional environment is a contextual factor that shapes a regulatory and normative environment, influencing firm behaviour. In particular, in this study, we refer to the institutionalisation level as a quality of the institutional environment that supports a free-competition market (Wang and Qian 2011; Hu and Sun 2018) and its impact on engagement with ESG criteria. In developing or transitional economies, such as China, the level of institutionalisation is important, as it changes competition regulations and market depth. Wang et al. (2018) used several dimensions to represent the concept, such as the level of development of the private sector, state intervention that indicates how large the free competition in a market is, or the presence of intermediaries (financial and legal services) that indicate the general level of sophistication of a market, up to the level of human capital availability (Wang et al. 2018).

After the signing of the Paris Agreement in 2016, many countries began to build an institutional environment that led firms to adopt sustainable development goals by adding ESG criteria to their industrial quality standards and strategies. Thus, financial and industrial associations can request that family firms follow ESG criteria when signing business contracts or developing strong business relationships (García-Sánchez et al. 2021). It has been confirmed that a firm with a high ESG score is favoured when it comes to gathering external financial resources (Kordsachia 2021; Ozdemir et al. 2021). The cost of debt for secondary market transactions, for example, seems lower for buildings that are environmentally certified (Eichholtz et al. 2019). Furthermore, in a more institutionalised environment, hiring nonfamily directors and fostering deeper collaboration with external suppliers can help a family business be recognised by financial institutions as trustworthy (Cordeiro et al. 2021). Since family ownership is more externally driven when it comes to persevering SEW endowments and adopting ESG criteria, any external pressure received from a more institutionalised environment may be beneficial. Therefore, we propose the following:

#### *Hypothesis 4a*

Institutionalisation positively moderates the relationship between family ownership and a firm’s ESG score.

Family control is more internally driven, and the adoption of ESG criteria concerns maintaining binding social ties for the family in charge of decisions; the institutional environment may therefore have a dissimilar moderating effect. First, when building a legal and institutional environment to protect the market and the investors’ interests, one of the primary considerations of the firm is to strengthen the independence and professionalisation of the board of directors and/or of supervisors, increasing mutual and restraint mechanisms between these bodies (García-Sánchez et al. 2020). However, hiring non-family employees to join the board of directors could be considered a threat to SEW endowment from an internal perspective, as the amount of control held by family members is reduced (Berrone et al. 2012; Kalm and Gomez-Mejia 2016). Generally, a more institutionalised environment is more incisive in trying to stimulate the adoption of ESG criteria (Ortas et al. 2019). These situations may not align with the goals of a family or its family members (Wang et al. 2020). An institutional environment can put pressure on a firm to issue industrial quality standards at a certain level (Dabić et al. 2016), forcing the interruption of collaborations with suppliers or partners who do not adopt ESG criteria (Mukandwal et al. 2020). This could break relationships and destroy family SEW endowments from an internal perspective. Therefore, whether family control has a positive influence (Hp2a) or negative influence (Hp2b) on ESG adoption, its impact appears to be reduced in an institutionalised environment. Thus, we propose the following hypothesis:

#### *Hypothesis 4b*

Institutionalisation negatively moderates the relationship between family control and a firm’s ESG score.

To summarise the overall research design, this study highlights the influence of family involvement, both in terms of family ownership and family control, on a firm’s ESC scores (Hp1 and Hp2a, b). We further test two moderation effects, namely market competition (Hp3a, b) and institutionalisation (Hp4a, b), as contextual factors that moderate the main relationships differently.

## Methodology

### Sample selection and data collection

The data used in this study have been drawn from a sample of listed family-owned firms in China that have been included in the ‘Growth Enterprise Market and Small and Medium Enterprise Board’ for all the period in the analysis (2014–2019). According to a PWC family business survey (2021),^1^ private owned companies contribute more than 60% of China’s GDP. Within this huge category, 85% of these firms are family firms. China is a very fervent environment for studying the impact of ESG criteria from all three angles, environmental, social, and governance. From an environmental perspective, China has made great efforts; first, signing and executing ‘the Paris Agreement’, implemented during the 21st Climate Change Conference, organised by the United Nations. Secondly, by setting a series of ‘double carbon’ objectives, i.e. carbon peaking by 2030 and carbon neutrality by 2060. From a societal perspective, Chinese culture has a long tradition surrounding the concept of *jia guo*. This is the intersection of nationalism and familism, which asserts that people and, in turn, companies are naturally orientated towards participating in philanthropic activities and taking social responsibilities (Chen 2019). From a governance perspective, more can be done. Most of Chinese companies are less than 30 years old, thus relatively young, and for this reason they usually adopt immature or simple corporate governance structures. For example, CEOs that also sit as chairmen of their boards of directors and/or this latter body that is mostly made up of family members. Simple corporate governance structures can benefit from accepting new development values, such as ESG criteria.

After excluding listed companies that (1) operate in the financial and insurance industry, (2) receive special treatment from stock exchanges, (3) have evident financial anomalies, and (4) change their actual controller during the sample period, the final sample included 4,098 observations (1,151 firms). Table 1 lists the sample distribution by industry, size, and province.

**Table 1:** Sample distribution

Panel A: Industry					
Industry	Number	Industry	Number	Industry	Number
Agriculture, forestry, animal husbandry and fishery	64	Wholesale and retail	83	Scientific research and technology services	37
Mining	26	Transport, storage and post	25	Water conservancy, environment and public facilities management	42
Manufacturing	3156	Information transmission, software and information technology services	373	Health and social work	13
Electricity, heat, gas and water production and supply	21	Real estate	29	Culture, sports and entertainment	54
Construction	121	Leasing and business services	54		

Panel B: Size					
Amounts of assets (Yuan)	Number	Amounts of assets (Yuan)	Number	Amounts of assets (Yuan)	Number
0.1 billion – 1 billion	501	1 billion – 10 billion	3210	> 10 billion	387

Panel C: Province					
Province	Number	Province	Number	Province	Number
Guangdong	967	Liaoning	63	Hainan	21
Zhejiang	663	Hebei	62	Gansu	21
Jiangsu	540	Hubei	60	Heilongjiang	17
Beijing	360	Jiangxi	40	Yunan	16
Shandong	281	Chongqing	32	Inner Mongolia	15
Shanghai	183	Tianjin	29	Guizhou	15
Fujian	137	Jilin	26	Tibet	7
Sichuan	128	Guangxi	26	Ningxia	5
Hunan	119	Shanxi	23	Qinghai	5
Henan	102	Xinjiang	22		
Anhui	92	Shanxi	21		

Data on corporate governance and financial conditions are collected from the China Stock Market and Accounting Research (CSMAR) database, while those on ESG are collected from the Wind database. We collected data between October 2020 and April 2021. Continuous variables have been winsorized, for values under 1% and over 99%, to avoid the influence of outliers.

### Variable measurements

The dependent variable is the ESG score of a family firm (*ESGscore*). The Wind database discloses the ESG rating results of four institutions: the Sino-Securities Index, FTSE Russell, SynTao Green Finance, and China Alliance of Social Value Investment. Of these, the ESG rating covers the most listed companies. Therefore, in this paper, we use its rating results to measure the ESG performance of family firms. The Sino-Securities Index divides the ESG rating results into nine categories, with the highest being ‘AAA’ and the lowest being ‘C’. In this paper, we convert these results into numbers: “AAA” equals 9, “C” equals 1 and so on.

The independent variables are family ownership (*f_ownership*) and family control (*f_control*) in a family firm. According to Anderson and Reeb (2003), the family ownership rights and the extent of influence that a family can exert on the firm (right of control) are usually different. This is mainly because family members often control a listed company through a pyramid structure. Based on the ownership structure charts provided by the CSMAR database, we calculate family members’ ownership and their right of control following Lin et al. (2011). Specifically, in the chain of one ownership structure chart, family ownership is measured by multiplying the proportions along the chain. A simple example is as follows: if a family owns a fraction of shares in company A, and this company possesses a fraction of shares in company B, which is a listed company of the analysis, then the overall family ownership in company B is the product of the quota of shares possessed in A and B. When there are two or more chains in the ownership structure chart, we calculate the ownership of each chain and add them together to arrive at the final value of the *f_ownership* variable.

In the chain of one ownership structure chart, the right of control is measured by the weakest link. If the family owns a fraction of firm A (a), and firm A continues to own a fraction of firm B (b), which is the listed company, then control in firm B is the minimum level among the two ownerships (mathematically: min (a, b)). When there are two or more chains in the ownership structure chart, we calculate the right of control on each chain and add them to arrive at the final value for *f_control*.

Two moderating variables are introduced into the models to test their effects. The first is the degree of market competition. Following Ammann et al. (2013), we use the Herfindahl–Hirschman Index (*hhi*) to measure the degree of market competition. This index is calculated by summing up the squared market shares of all firms in a given industry and dividing them by the square of the sum of all market shares. According to He (2012), we reverse the sign of the original index by multiplying it by − 1, so a larger index indicates a higher degree of competition. We compute the degree of market competition (*hhi*) as follows:

$$hhi = \mathop \sum \limits_{i = 1}^{n} \left( {\frac{{x_{i} }}{x}} \right)^{2}$$

where x is the amount of total sales in one industry, xi is the amount of sales of firm i and n is the number of firms in this industry.

The second moderation variable is the level of institutionalisation of the market in which a firm is operating (*ins*). This variable is measured using the *institutionalisation index* calculated at the province level for the different provinces in China. In particular, the index contains the following information (Wang et al. 2018, p. 171). (1) The ties and relationships that the government establishes with the market and specifically: i) the overall level of private resources allocated, ii) the reduction in the government’s golden allocation quota in private ownership, and iii) the reduction in the direct intervention of the government. (2) The level of development reached by the private initiative (i.e. the numbers and scale of private-controlled firms and how these firms are distributed across different industries). (3) The development of the product market, and specifically: (i) the extent to which retail commodities, means of production, and agricultural product prices are freely determined by the market and (ii) the reduction in the local protection in commodity markets). (4) The development of the market factor and specifically: (i) the development of financial markets (e.g. marketisation of credit fund allocation), (ii) human capital development (e.g. presence of qualified and intellectual labour force), and (iii) the development of the technology market (e.g. workforce turnover in technology markets). (5) The level of development of both intermediary institutions operating in the market and the legal system and protections. The specific parameters are: i) the number of intermediaries, the level of their service conditions, and the level of assistance provided by industry associations, ii) the fairness of legal services, measured by evaluating companies’ opinions on the fairness and efficiency of local law enforcement agencies, and iii) the level of protection of intellectual property, calculated as the number of patent applications approved by the average number of scientific and technological personnel present in the market). After ponderation, the five dimensions are summarised into an index for each province and for each year.

In terms of control variables, we used elements already considered significant to affect the ESG performance of family firms. Some variables are related to the financial characteristics of a firm, including (1) Tobin’s Q (*tobinQ*), calculated using a firm’s market value divided by the cost of replacement cost; (2) total asset turnover ratio (*assetturnover*), calculated using the total revenue divided by its total assets; (3) debt ratio (*lev*), measured as the ratio between the totals of liabilities and assets; (4) *company size* (*size*), measured by the natural logarithm of total assets and (5) *company age* (*age*), calculated using the focused year minus the year in which the firm was established, plus 1. The other variables concern corporate governance structures, including (1) the proportion of independent directors (*independent*), measured as the ratio between independent directors and the total number of them; (2) the shareholding ratio of the largest shareholder (*top1*), calculated as the largest ownership quota divided by the total number of shares; (3) the board size (*boardnumber*), indicated by the overall number of directors; and (4) the leadership structure (*dual*), which equals 1 if one person serves as the board chairman and CEO at the same time and 0 otherwise. In addition, we control for the fixed effect caused by the year selected (*year*) and the sector in which the firm operates (*industry*).

These variables are defined in Table A7 in the appendix.

### Models

This paper uses multivariate regression analysis to test these hypotheses. The following models are used to test the main effect of the hypotheses.

1 $$\begin{aligned} {\text{ESGscore}} = & {\upalpha } + {\upbeta }_{{1}} *{\text{f}}\_{\text{ownership}} + {\upbeta }_{{2}} *{\text{tobinQ}} + {\upbeta }_{{3}} *{\text{assetturnover}} + {\upbeta }_{{4}} *{\text{lev}} \\ & + {\upbeta }_{{5}} *{\text{size}} + {\upbeta }_{{6}} *{\text{age}} + {\upbeta }_{{7}} *{\text{independent}} + {\upbeta }_{{8}} *{\text{top1}} \\ & + {\upbeta }_{{9}} *{\text{boardnumber}} + {\upbeta }_{{{1}0}} *{\text{dual}} + {\upbeta }_{{{11}}} *\sum {{\text{Year}}} + {\upbeta }_{{{12}}} *\sum {{\text{Industry}}} \\ \end{aligned}$$

2 $$\begin{aligned} {\text{ESGscore}} = & {\upalpha } + {\upbeta }_{{1}} *{\text{f}}\_{\text{control}} + {\upbeta }_{{2}} *{\text{tobinQ}} + {\upbeta }_{{3}} *{\text{assetturnover}} + {\upbeta }_{{4}} *{\text{lev}} \\ & + {\upbeta }_{{5}} *{\text{size}} + {\upbeta }_{{6}} *{\text{age}} + {\upbeta }_{{7}} *{\text{independent}} + {\upbeta }_{{8}} *{\text{top1}} + {\upbeta }_{{9}} *{\text{boardnumber}} \\ & + {\upbeta }_{{{1}0}} *{\text{dual}} + {\upbeta }_{{{11}}} *\sum {{\text{Year}}} + {\upbeta }_{{{12}}} *\sum {{\text{Industry}}} \\ \end{aligned}$$

We then add the moderation effects with the interaction terms of *hhi* and *ins*:

3 $$\begin{aligned} {\text{ESGscore}} = & {\upalpha } + {\upbeta }_{{1}} *{\text{f}}\_{\text{ownership}} + {\upbeta }_{{2}} *{\text{hhi}} + {\upbeta }_{{3}} *{\text{hhi}}*{\text{f}}\_{\text{ownership}} + {\upbeta }_{{4}} *{\text{tobinQ}} \\ & + {\upbeta }_{{5}} *{\text{assetturnover}} + {\upbeta }_{{6}} *{\text{lev}} + {\upbeta }_{{7}} *{\text{size}} + {\upbeta }_{{8}} *{\text{age}} + {\upbeta }_{{9}} *{\text{independent}} + {\upbeta }_{{{1}0}} *{\text{top1}} \\ & + {\upbeta }_{{{11}}} *{\text{boardnumber}} + {\upbeta }_{{{12}}} *{\text{dual}} + {\upbeta }_{{{13}}} *\sum {{\text{Year}}} + {\upbeta }_{{{14}}} *\sum {{\text{Industry}}} \\ \end{aligned}$$

4 $$\begin{aligned} {\text{ESGscore}} = & {\upalpha } + {\upbeta }_{{1}} *{\text{f}}\_{\text{ownership}} + {\upbeta }_{{2}} *{\text{ins}} + {\upbeta }_{{3}} *{\text{ins}}*{\text{f}}\_{\text{ownership}} + {\upbeta }_{{4}} *{\text{tobinQ}} \\ & + {\upbeta }_{{5}} *{\text{assetturnover}} + {\upbeta }_{{6}} *{\text{lev}} + {\upbeta }_{{7}} *{\text{size}} + {\upbeta }_{{8}} *{\text{age}} + {\upbeta }_{{9}} *{\text{independent}} \\ & + {\upbeta }_{{{1}0}} *{\text{top1}} + {\upbeta }_{{{11}}} *{\text{boardnumber}} + {\upbeta }_{{{12}}} *{\text{dual}} + {\upbeta }_{{{13}}} *\sum {{\text{Year}}} + \beta_{{{14}}} *\sum {{\text{Industry}}} \\ \end{aligned}$$

5 $$\begin{aligned} {\text{ESGscore}} =\, & {\upalpha } + {\upbeta }_{1} *{\text{f\_ownership}} + {\upbeta }_{2} *{\text{hhi}} + {\upbeta }_{3} *{\text{ins}} + {\upbeta }_{4} *{\text{hhi}}*{\text{f\_ownership}} + {\upbeta }_{5} *{\text{ins}}*{\text{f\_ownership}} \\ & + {\upbeta }_{6} *{\text{tobinQ}} + {\upbeta }_{7} *{\text{assetturnover}} + {\upbeta }_{8} *{\text{lev}} + {\upbeta }_{9} *{\text{size}} + {\upbeta }_{10} *{\text{age}} + {\upbeta }_{11} *{\text{independent}} \\ & + {\upbeta }_{12} *{\text{top}}1 + {\upbeta }_{13} *{\text{boardnumber}} + {\upbeta }_{14} *{\text{dual}} + {\upbeta }_{15} *\sum {{\text{Year}}} + {\upbeta }_{16} *\sum {{\text{Industry}}} \\ \end{aligned}$$

6 $$\begin{aligned} {\text{ESGscore}} = \,& {\upalpha } + {\upbeta }_{{1}} *{\text{f}}\_{\text{control}} + {\upbeta }_{{2}} *{\text{hhi}} + {\upbeta }_{{3}} *{\text{hhi}}*{\text{f}}\_{\text{control}} + {\upbeta }_{{4}} *{\text{tobinQ}} \\ & + {\upbeta }_{{5}} *{\text{assetturnover}} + {\upbeta }_{{6}} *{\text{lev}} + {\upbeta }_{{7}} *{\text{size}} + {\upbeta }_{{8}} *{\text{age}} + {\upbeta }_{{9}} *{\text{independent}} + {\upbeta }_{{{1}0}} *{\text{top1}} \\ & + {\upbeta }_{{{11}}} *{\text{boardnumber}} + {\upbeta }_{{{12}}} *{\text{dual}} + {\upbeta }_{{{13}}} *\sum {{\text{Year}}} + {\upbeta }_{{{14}}} *\sum {{\text{Industry}}} \\ \end{aligned}$$

7 $$\begin{aligned} {\text{ESGscore}} = \,& {\upalpha } + {\upbeta }_{{1}} *{\text{f}}\_{\text{control}} + {\upbeta }_{{2}} *{\text{ins}} + {\upbeta }_{{3}} *{\text{ins}}*{\text{f}}\_{\text{control}} + {\upbeta }_{{4}} *{\text{tobinQ}} \\ & + {\upbeta }_{{5}} *{\text{assetturnover}} + {\upbeta }_{{6}} *{\text{lev}} + {\upbeta }_{{7}} *{\text{size}} + {\upbeta }_{{8}} *{\text{age}} + {\upbeta }_{{9}} *{\text{independent}} \\ & + {\upbeta }_{{{1}0}} *{\text{top1}} + {\upbeta }_{{{11}}} *{\text{boardnumber}} + {\upbeta }_{{{12}}} *{\text{dual}} + {\upbeta }_{{{13}}} *\sum {{\text{Year}}} + {\upbeta }_{{{14}}} *\sum {{\text{Industry}}} \\ \end{aligned}$$

8 $$\begin{aligned} {\text{ESGscore}} =\, & {\upalpha } + {\upbeta }_{{1}} *{\text{f}}\_{\text{control}} + {\upbeta }_{{2}} *{\text{hhi}} + {\upbeta }_{{3}} *{\text{ins}} + {\upbeta }_{{4}} *{\text{hhi}}*{\text{f}}\_{\text{control}} \\ & + {\upbeta }_{{5}} *{\text{ins}}*{\text{f}}\_{\text{control}} + {\upbeta }_{{6}} *{\text{tobinQ}} + {\upbeta }_{{7}} *{\text{assetturnover}} + {\upbeta }_{{8}} *{\text{lev}} \\ & + {\upbeta }_{{9}} *{\text{size}} + {\upbeta }_{{{1}0}} *{\text{age}} + {\upbeta }_{{{11}}} *{\text{independent}} + {\upbeta }_{{{12}}} *{\text{top1}} \\ & + {\upbeta }_{{{13}}} *{\text{boardnumber}} + {\upbeta }_{{{14}}} *{\text{dual}} + {\upbeta }_{{{15}}} *\sum {{\text{Year}}} + {\upbeta }_{{{16}}} *\sum {{\text{Industry}}} \\ \end{aligned}$$

## Results

### Descriptive statistics and correlation analysis

Table 2 contains the main descriptive statistics associated with the variables used in the regression models. The mean value of *the ESG score* is 6.186, which means that the ESG performance of family firms in our sample is relatively high. Its minimum and maximum values are 2 and 9, respectively. The mean values of *f_ownership* and *f_control* are 38.96 and 42.48, respectively. This shows that there is a difference between family member ownership and right of control. Considering the moderating variables, the mean value of *hhi* is -0.046, and its standard deviation is 0.067. This latter index shows quite a variance in the degree of market competition experienced by the family firms in our sample. The minimum and maximum values of *ins* are 0.710 and 10, respectively, which indicates that there are differences in the institutionalisation levels of family firms. After dealing with the outliers, the distributions of our control variables are as expected.

**Table 2:** Descriptive statistics

variable	N	mean	p50	sd	min	max
ESGscore	4098	6.186	6	0.925	2	9
f_ownership	4098	38.96	37.54	15.23	10.94	73.39
f_control	4098	42.48	41.98	14.74	14.26	74.96
hhi	4098	− 0.046	− 0.016	0.067	− 0.369	− 0.008
ins	4098	8.792	9.300	1.403	0.710	10
tobinQ	4098	2.706	2.224	1.580	1.047	9.826
assetturnover	4098	0.595	0.515	0.363	0.121	2.354
lev	4098	35.61	33.85	17.81	5.298	80.92
size	4098	21.78	21.72	0.892	20.03	24.15
age	4098	22.281	22	4.582	12	47
independent	4098	0.380	0.364	0.0530	0.333	0.571
top1	4098	32.96	31.41	12.24	11.91	65.46
boardnumber	4098	8.020	9	1.377	4	15
dual	4098	0.410	0	0.492	0	1

Table 3 shows the results of the correlation analysis. As indicated, the correlations between *ESGscore* and both *f_ownership* and *f_control* are positive and significant at the 1% level. Both family ownership and control are correlated with the firm’s ESG scores and performance. This gave us the opportunity to further test the hypothesis with the regression model. We also calculate the variance inflation factor (VIF). The maximum value of the VIF is 1.97, which indicates that there is no collinearity problem in our regression models. However, when the two independent variables are included in the same regression model, the VIF values of *f_ownership* and *f_control* are 6.26 and 8.02, respectively, which are relatively high. Therefore, we add these two variables to separate regression models.

**Table 3:** Correlation analysis

	ESGscore	f_ownership	f_control	hhi	ins	tobinQ	assetturnover	lev	size	age	independent	top1	boardnumber	dual
ESGscore	1													
f_ownership	0.052***	1												
f_control	0.048***	0.908***	1											
hhi	− 0.087***	0.096***	0.114***	1										
ins	0.018	0.034**	0.025	− 0.026*	1									
tobinQ	0.047***	− 0.050***	− 0.037**	− 0.190***	− 0.009	1								
assetturnover	0.028*	0.060***	0.111***	0.064***	0.089***	− 0.116***	1							
lev	− 0.094***	− 0.081***	− 0.065***	0.003	0.041***	− 0.309***	0.245***	1						
size	0.097***	− 0.095***	− 0.064***	0.025	0.049***	− 0.345***	0.160***	0.505***	1					
age	− 0.020	0.046***	0.030*	0.030*	0.054***	0.022	− 0.010	− 0.033**	− 0.057***	1				
independent	− 0.009	0.138***	0.103***	− 0.019	0.036**	0.065***	− 0.052***	− 0.047***	− 0.105***	− 0.023	1			
top1	0.021	0.579***	0.697***	0.055***	− 0.014	0.017	0.099***	− 0.032**	− 0.041***	− 0.011	0.092***	1		
boardnumber	0.041***	− 0.126***	− 0.079***	− 0.003	− 0.058***	− 0.081***	0.044***	0.053***	0.175***	0.059***	− 0.684***	− 0.066***	1	
dual	− 0.025	0.091***	0.050***	0.018	0.101***	0.022	− 0.008	− 0.035**	− 0.111***	− 0.015	0.137***	0.091***	− 0.129***	1

*p < 0.1, ** *p *< 0.05, *** *p *< 0.01

### Multivariate regression analysis

Table 4 shows the results of the multivariate regression analysis obtained using the OLS method and *f_ownership* as an independent variable. Model (1) shows that the *f_ownership* coefficient is positive and statistically significant at the 1% level, which means that a larger family members’ ownership quota increases the commitment of the adoption of the family firm to the ESG criteria, resulting a better ESG score. These results support Hypothesis H1. For the control variables, family firms with high Tobin’s Q, high total asset turnover ratio, and low debt ratio, as well as large size, are more likely to implement ESG practices. To test the moderating effect of a firm’s degree of market competition, an interaction term between *hhi* and *f_ownership* is added to the following model (Model 2). The interaction term coefficient is negative and statistically significant at the 1% level. These results indicate that the influence of *f_ownership* on the *ESGscore* is less significant in family firms with high degrees of competition compared to those with low degrees of competition. This is consistent with Hypothesis H3a. To test the moderating effect of the level of institutionalisation where a firm is located, an interaction term between *ins* and *f_ownership* is added to Model (3). Considering this interaction effect and its coefficient, the results are not statistically significant; thus, Hypothesis H4a is rejected. In Model (4), we add the interaction terms between *hhi* and *f_ownership* and between *ins* and *f_ownership*. As indicated, the results remain the same.

**Table 4:** Regression analysis results: Using *f_ownership* as independent variable

	(1)	(2)	(3)	(4)
	ESGscore	ESGscore	ESGscore	ESGscore
f_ownership	0.005***	0.003**	0.011*	0.009
	(4.11)	(2.51)	(1.89)	(1.63)
hhi		− 0.071		− 0.072
		(− 0.10)		(− 0.11)
f_ownership × hhi		− 0.038***		− 0.038***
		(− 2.75)		(− 2.75)
ins			0.034	0.033
			(1.22)	(1.21)
f_ownership × ins			− 0.001	− 0.001
			(− 1.08)	(− 1.11)
tobinQ	0.035***	0.032***	0.034***	0.031***
	(3.10)	(2.81)	(2.98)	(2.68)
assetturnover	0.144***	0.145***	0.140***	0.141***
	(3.30)	(3.33)	(3.18)	(3.22)
lev	− 0.010***	− 0.010***	− 0.010***	− 0.010***
	(− 9.91)	(− 9.90)	(− 9.90)	(− 9.89)
size	0.216***	0.218***	0.214***	0.216***
	(10.92)	(11.05)	(10.77)	(10.90)
age	− 0.003	− 0.002	− 0.003	− 0.002
	(− 0.80)	(− 0.63)	(− 0.81)	(− 0.62)
independent	0.417	0.435	0.427	0.445
	(1.16)	(1.21)	(1.18)	(1.23)
top1	− 0.002	− 0.002	− 0.002	− 0.002
	(− 1.17)	(− 1.30)	(− 1.14)	(− 1.27)
boardnumber	0.032**	0.032**	0.033**	0.033**
	(2.23)	(2.29)	(2.29)	(2.35)
dual	− 0.020	− 0.022	− 0.020	− 0.021
	(− 0.69)	(− 0.74)	(− 0.69)	(− 0.73)
Year	Yes	Yes	Yes	Yes
Industry	Yes	Yes	Yes	Yes
_cons	1.197**	0.928*	0.948*	0.681
	(2.43)	(1.85)	(1.78)	(1.25)
N	4098	4098	4098	4098
Adj− R^2^	0.073	0.076	0.073	0.076
F	12.892	12.701	11.930	11.930

*p < 0.1, ** *p *< 0.05, *** *p *< 0.01. T values are reported in brackets

**Fig. 1:**
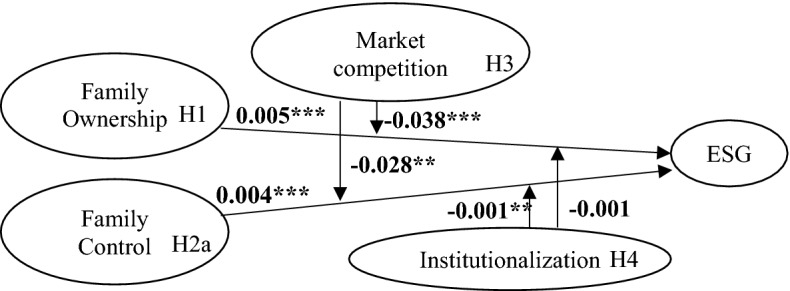
Results

Similarly, Table 5 shows the results of the multivariate regression analysis obtained using *f_control* as the independent variable. Model (1) shows the *f_control* coefficient as positive and significant (1% level). Therefore, the higher the right of control results in a better ESG performance of the family firm. Thus, H2a is supported, while H2b is rejected. To test the moderating effect of a firm’s degree of market competition, an interaction term between *hhi* and *f_control* is added to Model (2). As indicated, this interaction term is significant (5% level) and negative. In other words, the influence of *f_control* on *ESGscore* is less significant in family firms with high degrees of competition compared to those with low degrees of competition, which is consistent with Hypothesis H3b. To test the moderating effect of the level of institutionalisation where a firm is located, an interaction term between *ins* and *f_control* is added to Model (3). As for the previous interaction term, this coefficient is significant (5% level) and negative. Thus, when the level of institutionalisation of the environment increases, the influence of *f_control* on the *ESGscore* will be weakened. This is consistent with Hypothesis H4b. In Model (4), we add interaction terms between *hhi* and *f_control* and between *ins* and *f_control*. As indicated, the results remain the same. The overall results are summarised in Fig. 1.

**Table 5:** Regression analysis results: Using *f_control* as independent variable

	(1)	(2)	(3)	(4)
	ESGscore	ESGscore	ESGscore	ESGscore
f_control	0.004***	0.003**	0.017***	0.016***
	(3.31)	(2.20)	(2.87)	(2.69)
hhi		− 0.337		− 0.353
		(− 0.47)		(− 0.50)
f_control × hhi		− 0.028**		− 0.028**
		(− 2.05)		(− 2.03)
ins			0.067**	0.067**
			(2.23)	(2.24)
f_control × ins			− 0.001**	− 0.001**
			(− 2.16)	(− 2.21)
tobinQ	0.033***	0.030***	0.031***	0.027**
	(2.91)	(2.60)	(2.70)	(2.37)
assetturnover	0.137***	0.137***	0.130***	0.131***
	(3.12)	(3.14)	(2.96)	(2.98)
lev	− 0.010***	− 0.010***	− 0.010***	− 0.010***
	(− 9.93)	(− 9.95)	(− 9.89)	(− 9.92)
size	0.213***	0.216***	0.209***	0.212***
	(10.79)	(10.96)	(10.55)	(10.71)
age	− 0.002	− 0.002	− 0.002	− 0.002
	(− 0.71)	(− 0.56)	(− 0.67)	(− 0.51)
independent	0.437	0.461	0.445	0.469
	(1.21)	(1.28)	(1.23)	(1.30)
top1	− 0.002	− 0.002	− 0.002	− 0.002
	(− 1.25)	(− 1.25)	(− 1.25)	(− 1.26)
boardnumber	0.030**	0.031**	0.031**	0.032**
	(2.09)	(2.16)	(2.18)	(2.25)
dual	− 0.014	− 0.017	− 0.013	− 0.015
	(− 0.50)	(− 0.58)	(− 0.45)	(− 0.51)
Year	Yes	Yes	Yes	Yes
Industry	Yes	Yes	Yes	Yes
_cons	1.268***	0.963*	0.774	0.464
	(2.58)	(1.92)	(1.44)	(0.85)
N	4098	4098	4098	4098
Adj-R^2^	0.071	0.074	0.072	0.075
F	12.653	12.354	11.963	11.730

*p < 0.1, ** *p *< 0.05, *** *p *< 0.01. T values are reported in brackets

We further validated our results with some robust tests. There may be systematic differences related to the ESG performance between family- and non-family-owned companies. To avoid this sample selection bias, this paper uses the propensity score matching method to construct a new sample. In particular, the original sample is divided into a treatment group (high family ownership) and a control group (low family ownership) according to the median of *f_ownership*. The 1:1 nearest-neighbour matching process is carried out based on variables including *size*, *lev*, *Year,* and *Industry*. We then run the regressions using the matching sample. As shown in Table 6, the results remain robust.

**Table 6:** Robustness tests (PSM method)

	(1)	(2)
	ESGscore	ESGscore
f_ownership	0.005**	
	(2.32)	
f_control		0.005**
		(2.25)
tobinQ	0.041*	0.040
	(1.65)	(1.62)
assetturnover	0.235***	0.231***
	(2.72)	(2.67)
lev	− 0.009***	− 0.009***
	(− 4.58)	(− 4.54)
size	0.184***	0.181***
	(4.65)	(4.58)
age	0.010*	0.011*
	(1.71)	(1.74)
independent	1.362*	1.407**
	(1.90)	(1.96)
top1	− 0.004	− 0.005
	(− 1.22)	(− 1.44)
boardnumber	0.084***	0.082***
	(2.87)	(2.81)
dual	− 0.064	− 0.060
	(− 1.09)	(− 1.03)
Year	Yes	Yes
Industry	Yes	Yes
_cons	0.306	0.339
	(0.31)	(0.34)
N	1164	1164
Adj-R^2^	0.077	0.077
F	4.586	4.573

*p < 0.1, **p < 0.05, ***p < 0.01. T values are reported in brackets

## Discussion and conclusions

### Discussions

After analysing 4,098 observations of family-owned listed firms, this study finds that both family ownership and family control are positively related to ESG scores. As suggested, in driving the adoption of ESG criteria, both family ownership and family control are negatively moderated by market competition. To some extent, in contrast to our hypothesis, only the influence of family control on the ESG score is affected (negatively) by the level of institutionalisation. Accordingly, several interesting topics for discussion have arisen. The results of Hypotheses 1 and 2a are in line with the tradition in governance studies to study the mechanisms of the impact of corporate governance on ESG scores. Shareholder values and goals are vital in influencing goals related to firms’ strategies and operations of firms (Friede 2019). We chose family ownership and control as specific types of family involvement and uncover positive stimulation of ESG scores for both. This can be explained by reference to the SEW theory, in that family identification and renewal of family bonds favour a family’s propensity to set goals with a long-term orientation and its desire to assume social responsibility for satisfying stakeholders (Tiberius et al. 2021). For Hp1, the preservation of the value of a shareholder’s quota can be considered a possible factor that explains why family ownership leads to higher ESG scores (Benz et al. 2021). As discussed, the preservation of this value and thus of the family investment, especially considering large family businesses, must also satisfy external stakeholders. This can be achieved by adopting sustainable and fair practices and a balanced governance model, which results in better ESG scores. Instead, in relation to Hp2 (a, b), a shareholder coalition with a large control quota can develop a strong sense of personal identity with the reputation of a company, setting long-term goals when making decisions (O'Rourke et al. 2003).

In our particular case, a positive relationship is found between family control and ESG criteria, confirming Hp2a. However, at the theoretical level, a negative relationship (Hp2b) cannot be completely ignored. A positive relationship is likely to exist as a result of an internally driven motivation (e.g. personal/organisational values and ideas) that is aligned with ESG criteria. In the Chinese context, this interpretation may be congruent with cultural explanations, and the *jia guo* concept can be used to support this claim. *Jia guo* refers to the general sentiment of people influenced by Chinese culture and tradition that exalts the intersection between nationalism and familism. This concept leads individuals to be proud of their social surroundings, both in their close environment (familism) and in society more broadly (nationalism). These elements are derived from the collectivistic nature of Chinese and many other cultures in the Far East. Family-controlled firms, although less involved with external stakeholders, still strongly identify their family reputation with the business success of their companies (Berrone et al. 2012). This means that Chinese family-controlled companies will gladly engage with socially and environmentally friendly practices (Chen 2019) for internal congruence with the set of culturally embedded in their families. In other words, the reputation of a family is an important and internally orientated force that drives the adoption of ESG criteria by family firms (García-Sánchez et al. 2020). (García-Sánchez et al. 2020). For this reason, these results may be quite idiosyncratic to the Chinese context, and generalisations must be made carefully.

The results of Hypotheses 3a and 3b are consistent with those obtained by recent SEW studies that stress the tension and trade-off between economic and non-economic goals. This perspective is enriched by including ESG criteria in the discussion. On the one hand, this result supports the idea that business-owning families are challenged in their decision-making processes when evaluating ESG adoption, regardless of the type of involvement, both family ownership and control. When competition is greater, performance can be affected, and for this reason, families can neglect ESG practices in favour of dedicating more resources to regaining competitiveness (Ratten et al. 2021).

Finally, the results of Hypotheses 4b confirm that a stronger institutional environment can have a significant moderating effect, thus restraining the possibility for a family control to influence the adoption and performance. No moderating effect is found for family ownership, opposing our hypothesis (Hp4a). On the one hand, the institutional environment is found to be an efficient instrument when it comes to reducing the power of the power of large coalitions (Peng et al. 2018). The existence of intermediaries and working unions may restrict the decision-making freedom of a family. On the other hand, the current institutional environment fails to recognise that family control may also have a positive effect on adopting ESG criteria.

### Contributions

These interesting findings reveal several theoretical contributions and implications.

For its theoretical contributions, this study contributes both to the study of ESG and to research on family firms. The negative moderating effect of market competition that this research has discovered enriches ESG studies and draws attention to the tension between ESG criteria and firm performance. Unlike traditional studies that have focused on firm performance, market competition stresses comparisons between firms. Although many studies have confirmed a positive connection between high ESG scores and better firm performance, this higher expectation of performance can be undermined by the shadow of competition. Especially today, sluggish market demand and rising prices of raw materials are pushing shareholders to rethink environmental management, as they begin to question whether ESG criteria are a liability or an investment in the long-term generation of profit. This is a key question in ESG studies. The tension between ESG criteria and firm performance should be further studied to enable us to fully understand how expectations about (a dire) competition may impact ESG adoption. On the contrary, distinguishing between family control and family ownership enriches traditional discussions on differences between family firms, indicating that the level and type of family involvement should evolve and fit with the evolutionary stage of a family firm (Chirico et al. 2011). Future studies should pay more attention to this and differentiate between family firms according to their degree of family involvement. This would establish more reliable considerations with respect to the adoption of ESG criteria.

The negative effects of market competition and institutional environments reveal regulatory implications from two perspectives. On the one hand, ESG criteria are still not fully accepted in emerging and transitional economies, and financial institutions and investors still consider ESG as a cost. That is, regulatory and informal institutions must promote the construction of formal institutions and foster understanding of environmentally friendly investments. Finally, this study offers implications for policymakers, as it reveals that substantial shareholders do not always expropriate the interests of small shareholders when making decisions about environmental management (Cordeiro et al. 2021). Further debate is therefore needed to establish ways in which institutions can detect virtuous behaviours of this kind.

### Limitations of the study and related future research directions

In addition to its merits, this study also has limitations that require future research. The first limitation concerns the study’s research design. Market competition was used to establish tension between the adoption of ESG criteria and firm competitiveness. A future research direction could be the assessment of triggers and barriers with regard to the adoption of ESG criteria. Most Chinese family firms do not have a sophisticated corporate governance structure. Their conditions may therefore be unfavourable to listed firms, as stock exchange market regulations and other financial institutions emphasise formal regulations and information symmetry between inside shareholders and outside investors. As a transitional economy, China does not have a sophisticated institutional system to support firms’ adoption of ESG criteria, and many preliminary regulations and market institutions have only just begun to be issued. A final limitation that presents a future research direction is related to the cultural idiosyncrasy of the study. The results may be culturally bound, and, as such, when applied to other contexts outside of China, they should be carefully reviewed. For this reason, this research design should be further validated across different nations to truly understand what drives family firms’ adoption of ESG criteria.

## Appendix

See Table

**Table 7:** Definitions of variables

Variable types	Variables	Definitions
Dependent variable	ESGscore	Sino-Securities Index’s ESG rating results, “AAA” equals 9, “C” equals 1, others and so on
Independent variable	f_control	Measured by the weakest link in the chain of one ownership structure chart
	f_ownership	Measured by multiplying the proportions along the ownership chain
Moderating variables	hhi	Measured by the Herfindahl–Hirschman Index (hhi) to measure market competition degree. This index is calculated by summing up the squared market shares of all firms in a given industry and divide it by the square of the sum of all market shares
	int	Measured by the total institutionalisation index of different provinces in China disclosed by the “Marketization Index of China’s Provinces: NERI report” (Wang et al. 2018)
Control variables	tobinQ	Firm’s market value divided by asset replacement cost
	assetturnover	Firm’s total revenue divided by total asset
	lev	Total liabilities divided by total assets
	size	Natural logarithm of a firm's total assets
	age	using the focused year minus the year when the firm was established, then plus 1
	independent	Proportion of independent directors in the board of directors
	top1	The number of shares held by the largest shareholder divided by the total number of shares
	boardnumber	Total number of directors
	dual	Dummy variable, it equals to 1 if one person serves as chairman and CEO at the same time. Otherwise, it equals to 0
	year	4 year dummy variables
	industry	13 industry dummy variables

7

## Footnotes

1 https://www.pwccn.com/zh/services/entrepreneurial-and-private-business/private-family-business-services/global-family-business-survey-2021.html.
